# Modeling the role of gap junctions between excitatory neurons in the developing visual cortex

**DOI:** 10.1371/journal.pcbi.1007915

**Published:** 2021-07-06

**Authors:** Jennifer Crodelle, David W. McLaughlin

**Affiliations:** 1 Middlebury College, Middlebury, Vermont, United States of America; 2 Courant Institute of Mathematical Sciences, NYU, New York, New York, United States of America; 3 Center for Neural Science, NYU, New York, New York, United States of America; 4 Neuroscience Institute of NYU Langone Health, New York, New York, United States of America; 5 New York University Shanghai, Shanghai, China; Inria, FRANCE

## Abstract

Recent experiments in the developing mammalian visual cortex have revealed that gap junctions couple excitatory cells and potentially influence the formation of chemical synapses. In particular, cells that were coupled by a gap junction during development tend to share an orientation preference and are preferentially coupled by a chemical synapse in the adult cortex, a property that is diminished when gap junctions are blocked. In this work, we construct a simplified model of the developing mouse visual cortex including spike-timing-dependent plasticity of both the feedforward synaptic inputs and recurrent cortical synapses. We use this model to show that synchrony among gap-junction-coupled cells underlies their preference to form strong recurrent synapses and develop similar orientation preference; this effect decreases with an increase in coupling density. Additionally, we demonstrate that gap-junction coupling works, together with the relative timing of synaptic development of the feedforward and recurrent synapses, to determine the resulting cortical map of orientation preference.

## Author summary

Gap junctions, or sites of direct electrical connections between neurons, have a significant presence in the cortex, both during development and in adulthood. Their primary function during either of these periods, however, is still poorly understood. In the adult cortex, gap junctions between local, inhibitory neurons have been shown to promote synchronous firing, a network characteristic thought to be important for learning, attention, and memory. During development, gap junctions between excitatory, pyramidal cells, have been conjectured to play a role in synaptic plasticity and the formation of cortical circuits. In the visual cortex, where neurons exhibit tuned responses to properties of visual input such as orientation and direction, recent experiments show that excitatory cells are coupled by gap junctions during the first postnatal week and are replaced by chemical synapses during the second week. In this work, we explore the possible contribution of gap-junction coupling during development to the formation of chemical synapses between the visual cortex from the thalamus and between cortical cells within the visual cortex. Specifically, using a mathematical model of the visual cortex during development, we identify the response properties of gap-junction-coupled cells and their influence on the formation of the cortical map of orientation preference.

## Introduction

Gap junctions (GJs), or sites of direct electrical coupling between neurons, are present in the primary visual cortex (V1) at many stages of life, from infancy to adulthood. In the adult cortex, gap-junction coupling among local, inhibitory cells has been shown to promote synchrony, a property underlying many cognitive processes such as learning and memory [1, 2]. Though GJs have been measured between excitatory, pyramidal neurons in the adult cortex [3], there are very few experiments and the couplings were found to be very rare; consequently, their function remains unclear [4, 5]. Recent experiments show that pyramidal cells are coupled by GJs during the first postnatal week of development [6, 7], a time at which chemical synapses are highly plastic and are just beginning to develop [8], leading to a question about a potential relationship between GJ coupling and the development of V1 neuron response properties.

One example of a response property of cells in V1 is orientation preference (OP), where neurons preferentially respond to the orientation angle of a visual stimulus. In some higher-level mammals such as monkeys and cats, the visual cortex contains an ordered map of the orientation preference of each neuron, where cells preferring similar angles reside close to one another [9, 10]. In rodents, however, the map of orientation preference appears random and disordered, with little correlation between preferred orientation and location in cortical space [11, 12]. Despite the seemingly random lateral (within layer) distribution of OPs in the visual cortex of mice, radially-distributed clonally-related cells show similar stimulus feature selectivity [8], as well as preferential synaptic connectivity with fellow sister cells [13]. Each of these characteristics, measured in the adult cortex, relies on gap-junction coupling between sister cells during the first postnatal week [6, 7].

**Fig 1:**
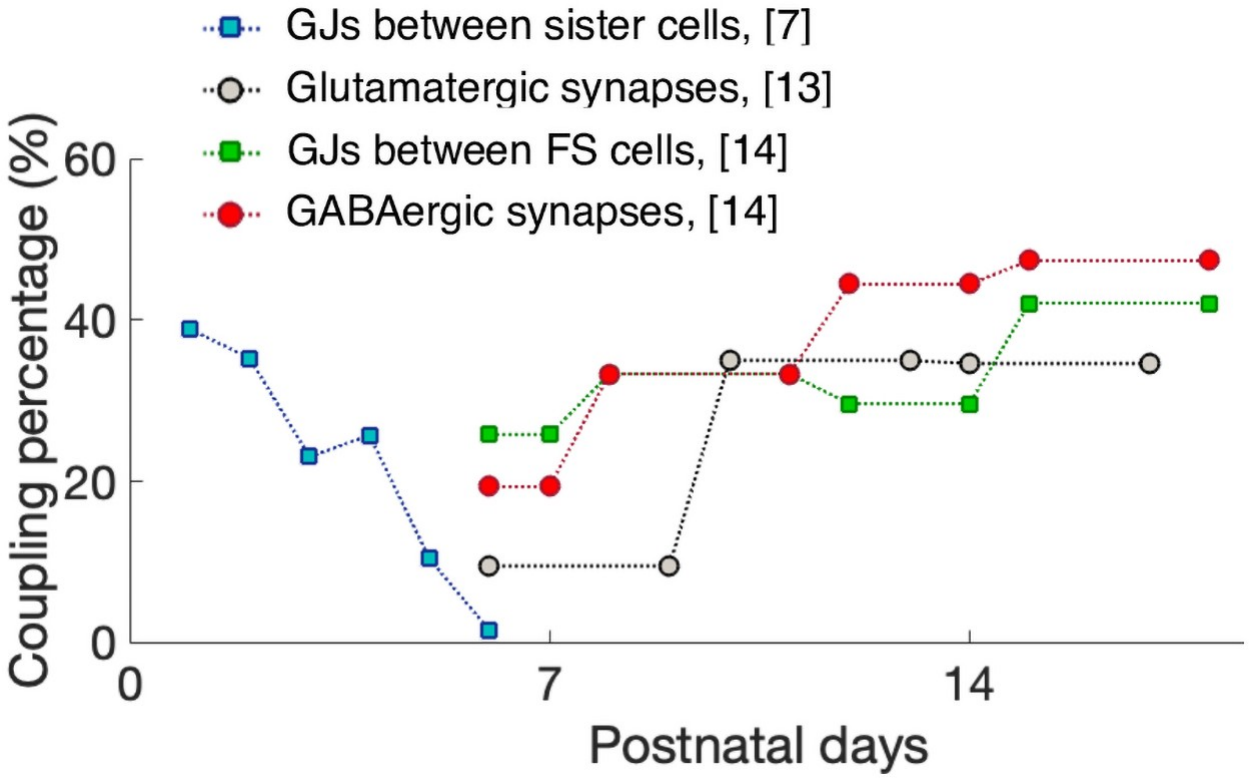
Different percentages of couplings over the first few postnatal weeks. The coupling probability for glutamatergic synapses is measured for radially-aligned sister cells only, while the GJs and GABAergic synapses are measured between fast-spiking (FS) cells. The experimental data was often reported as an average over several days, represented here as horizontal lines.

In particular, despite the lack of synaptic couplings during the first postnatal week (P0-P6), radially-aligned sister cells preferentially form synapses with an average probability of 36% in the second postnatal week [13]. In comparison, neighboring (also radially-aligned) non-sister cells are coupled with an average probability of about 6.3% (averaged over P10 to P17). Additionally, sister cells are preferentially coupled by GJs during the first postnatal week (28.2% for sister cells compared to 2.6% for non-sister cells, averaged over P1 to P6), with the probability of GJ connectivity decreasing steadily over the course of the first week (38.9% at P1 to ∼10% at P6) [7]. The strength of this GJ, as measured by the coupling coefficient (ratio of the amplitude of the response in the coupled cell to the response in the injected cell) is 5.7% for sister cells and 1.2% for non-sister cells (averaged over P1 to P6). This strength also decreases over the course of the first week (from 7.4% at P1 to 2.3% at P6) [7]. The black circles and blue squares in Fig 1 show the synaptic and GJ coupling percentage of excitatory sister cells, respectively, over the first few postnatal weeks.

Among inhibitory cells, GABAergic synapses and GJs form simultaneously beginning at the start of the second postnatal week [14] (in contrast to the pyramidal cells where GJs precede chemical synapses). Specifically, no GABAergic synapses or GJs are detected between FS cells from P3-P5, with the exception that one functional GABAergic synapse (out of 13 tested pairs) was detected at P5 [14]. No recordings were performed before P3. Therefore, we determine that both GJ and synaptic coupling among FS cells are absent during the first postnatal week and grow during the second postnatal week, as shown by the red circles and green squares in Fig 1A for synaptic and GJ coupling, respectively.

Blocking the GJ between sister cells during the first postnatal week leads to a reduction in the probability of synaptic coupling among sister cells (from 26% to 9.8% averaged over the second week [7]), demonstrating that GJ coupling during the first postnatal week is critical to the correct circuit formation in adult mice. Additionally, excitatory cells that share a similar OP have an increased likelihood to also be synaptically coupled [15]. GJ coupling may also play a role in stimulus feature selectivity, such as orientation preference, as about 59% of radially-aligned sister cells have similar OPs (difference in preferred angle less than 30°), while neighboring non-sister cells exhibit a difference in OP distribution that was not significantly different from the uniform distribution [6]. When GJs are blocked during the first postnatal week, the effect was destroyed; the distribution of OP difference for sister cells was no longer significantly different from the uniform distribution or the non-sister cell distribution.

Our aim in this work is to better understand how the developmental timeline, including GJ-coupling among sister cells, might affect properties of synaptic plasticity such as the formation of random or disordered OP maps. We develop a simple mathematical model as a conceptual realization of a local patch of mouse V1 during the first two postnatal weeks of development. Our model includes spike timing-dependent plasticity (STDP) of the feedforward synapses from LGN to V1 during the first postnatal week, together with STDP plasticity of the cortical-cortical recurrent excitatory synapses within V1 during the second postnatal week. Using this model, we reproduce experimentally-measured properties of GJ-coupled sister cells, such as a shared OP and preferential synaptic connectivity, and demonstrate that, during the first postnatal week, the OP of GJ-coupled cells develops faster than the OP of those cells that were not GJ-coupled. This increased learning rate results in more selectivity of the GJ-coupled sister cells than non-coupled cells at a time when synapses within V1 are beginning to form, proposing a mechanism for the “salt-and-pepper” random OP map observed in mice. We also identify mechanisms by which this OP map can become ordered as observed in higher-level mammals, further supporting our proposed mechanism for the development of disordered OP maps.

## Methods and models

For our mathematical model, we utilize existing frameworks for modeling the formation of input preference as in [16], but include more realistic spike-timing-dependent plasticity (STDP) rules for the visual cortex as in [17, 18] together with inhibitory plasticity as in [19]. Parameters for the model were extracted from various sources with the goal to keep the neurons’ behavior and firing rate as biologically relevant as possible, while maintaining stable and competitive plasticity rules.

### The mathematical model

We consider 1000 feedforward synapses, representing input from LGN to the visual cortex, coupled to each cell in our model neuronal network of either 400 or 256 cortical cells. Neurons are organized on a square grid with periodic boundary conditions (i.e., neuron 1 is adjacent to neuron 2 and 20, as well as neuron 21 and 381). The cortical neurons are randomly assigned to be excitatory with 80% probability or inhibitory with 20% probability. The subthreshold voltage of the *i*th cortical neuron of type *Q* = {*E*, *I*} is described using the leaky integrate-and-fire equation as follows

$$\begin{array}{c} \tau_{m}\frac{dv_{Q}^{i}}{dt}=-\left(v_{Q}^{i}-v_{l}\right)-g_{QE}^{i}\left(t\right)\left(v_{Q}^{i}-v_{E}\right)-g_{QI}^{i}\left(t\right)\left(v_{Q}^{i}-v_{I}\right)-g_{c,Q}\sum_{j}\left(v_{E}^{i}-v_{E}^{j}\right), \end{array}$$ (1)

where *τ*_*m*_ = 20 ms, *v*_*l*_ = −60 mV, *v*_*E*_ = 0 mV, and *v*_*I*_ = −80 mV as in [16]. Once the voltage reaches a threshold of −45 mV, the neuron is said to have spiked, the spike time is recorded, and the voltage is reset to −60 mV. Gap junctions are included only among excitatory neurons, such that the conductance term *g*_*c*,*Q*_ takes on a nonzero value *g*_*c*_ for *Q* = *E* and zero for *Q* = *I*, and are incorporated into the model through a direct resistive term where $v_{E}^{j}$ is the voltage of the *j*th pre-junctional neuron; see the last term in Eq (1). In addition, to model the spikelet induced in the post-junctional cell in response to an action potential in the pre-junctional cell, a 1 mV instantaneous jump in voltage of the post-junctional cell is included, as in previous models [20, 21].

The cortical synaptic conductances are modeled as having instantaneous rise times and exponential decay at each received spike time so that the excitatory and inhibitory conductance traces, respectively, follow the equations

$$\begin{array}{cc} \sigma_{E}\frac{dg_{QE}^{i}}{dt} & =-g_{QE}^{i},\;\;\;\text{where}\;\;g_{QE}^{i}\to g_{QE}^{i}+{\bar{g}}_{QE}^{i}\;\;\text{at}\;\text{each}\;\text{excitatory}\;\text{presynaptic}\;\text{spike}\;\text{time}\;\\ \sigma_{I}\frac{dg_{QI}^{i}}{dt} & =-g_{QI}^{i},\;\;\;\text{where}\;\;g_{QI}^{i}\to g_{QI}^{i}+{\bar{g}}_{QI}^{i}\;\;\;\;\text{at}\;\text{each}\;\text{inhibitory}\;\text{presynaptic}\;\text{spike}\;\text{time}\; \end{array}$$

where the neuron type of the postsynaptic cell is represented by *Q* = {*E*, *I*}, *σ*_*E*_ = 11 ms and *σ*_*I*_ = 15 ms. Note that the synaptic conductances have been normalized by the leakage conductance and are thus unit-less. The maximal excitatory conductance strength, ${\bar{g}}_{QE}^{i}$, and inhibitory conductance strength, ${\bar{g}}_{QI}^{i}$, can each take one of the following values: {${\bar{g}}_{EE}^{i},\;{\bar{g}}_{IE}^{i}$} and {${\bar{g}}_{EI}^{i},\;{\bar{g}}_{II}^{i}$} where the subscript *XY* denotes the direction of coupling from *Y* to *X*. We implement an absolute maximum on all excitatory synapses at $g_{E}^{\text{max}}$ and on all inhibitory synapses at $g_{I}^{\text{max}}$. In this model, the conductances ${\bar{g}}_{II}^{i}={\bar{g}}_{II}$ and ${\bar{g}}_{IE}^{i}={\bar{g}}_{IE}$ are held constant at $0.3g_{I}^{\text{max}}$ and $0.1g_{E}^{\text{max}}$, respectively, for all cells, while ${\bar{g}}_{EE}^{i}\left(t\right)$ and ${\bar{g}}_{EI}^{i}\left(t\right)$ are plastic, changing with rules defined in the following subsection.

The external drive to the cortical network has two components: synaptic input from the LGN and a generic background drive to all cells. This external drive affects the excitatory conductance, $g_{QE}^{i}\left(t\right)$, as follows

$$\begin{array}{c} g_{QE}^{i}\left(t\right)\to g_{QE}^{i}\left(t\right)+{\bar{g}}_{\text{LGN}}^{i}\left(t\right)\;\;\text{at}\;\text{each}\;\text{feedforward}\;\text{LGN}\;\text{synapse}\;\text{spike}\;\text{time}\\ g_{QE}^{i}\left(t\right)\to g_{QE}^{i}\left(t\right)+{\bar{g}}_{\text{back}}^{i}\;\;\text{at}\;\text{each}\;\text{background}\;\text{spike}\;\text{time}, \end{array}$$

where ${\bar{g}}_{\text{LGN}}^{i}\left(t\right)$ is plastic, but ${\bar{g}}_{\text{back}}^{i}$ is constant at 0.02. The spike times of the background drive are generated from a Poisson process with rate 0.5 Hz. Each feedforward LGN synapse generates spikes using a Poisson spike train with a firing rate that depends on its own label. Specifically, the firing rate of LGN synapse labeled *a* in response to a stimulus at input location *s* is given by

$$\begin{array}{c} r_{a}=R_{0}+R_{1}(e^{-{\left(s-a\right)}^{2}/2\sigma^{2}}+e^{-\left(s+1000-a^{2}\right)/2\sigma^{2}}+e^{-\left(s-1000-a^{2}\right)/2\sigma^{2}}), \end{array}$$

as in [16], where *R*_0_ = 5 Hz, *R*_1_ = 20 Hz, and *σ* = 80. Input to these synapses consists of brief presentations of a uniformly randomly-chosen stimulus index (*a* in above equation) for a period of time that is chosen from an exponential distribution with mean 20 ms. All cortical cells receive input from LGN synapses with a 25% probability. While the inhibitory cells have a constant LGN feedforward synaptic strength randomly chosen uniformly between $\left[0,0.18g_{\text{LGN}}^{\text{max}}\right]$, the excitatory cells contain a plastic or variable strength, ${\bar{g}}_{\text{LGN}}^{i}\left(t\right)$.

### Plasticity rules

Feedforward LGN synapses to excitatory cortical cells, as well as the recurrent synapses between cortical excitatory cells, are plastic, with the strength of their connection, ${\bar{g}}_{\text{LGN}}^{i}\left(t\right)$ and ${\bar{g}}_{EE}^{i}\left(t\right)$, respectively, obeying the minimal triplet rule for the visual cortex [17]. We use the STDP triplet rule rather than the standard pre-post STDP rule that was used in [16] because we wish to reproduce the realistic bi-directional coupling that develops in the visual cortex of mice, a feat which cannot be accomplished with the pair-based STDP rules. In addition, experiments show that the STDP curves exhibited by pyramidal cells in the visual cortex of mice do not follow the typical slightly-asymmetric shape of potentiation and depression as in [22], but rather potentiation only occurs if the post-synaptic neuron had recently fired a spike of its own [17, 23]—a property that is captured by the triplet rule.

For each pre- and post-synaptic spike, the strength of the synapse from the pre- to post-synaptic cell, $\bar{g}\left(t\right)$, (dropping the *EE* subscript) is updated via the equations

$$\begin{array}{c} \bar{g}\left(t\right)\to \bar{g}\left(t\right)-o_{1}\left(t\right){\text{A}}_{\text{LTD}}\left(t\right)\;\;\text{if}\;t=t^{\text{PRE}}, \end{array}$$ (2)

$$\begin{array}{c} \bar{g}\left(t\right)\to \bar{g}\left(t\right)+r_{1}\left(t\right)o_{2}\left(t-\epsilon \right)A_{\text{LTP}}\;\;\text{if}\;t=t^{\text{POST}}, \end{array}$$ (3)

where *A*_*LTD*_(*t*) and *A*_*LTP*_ represent the strength of depression and potentiation, respectively. The tracer variables each follow the equation

$$\begin{array}{c} \frac{dx(t)}{dt}=-\frac{x(t)}{\tau_{x}}\;\;\text{for}\;\;x=r_{1},\;o_{1},\;o_{2}, \end{array}$$ (4)

where *r*_1_(*t*) represents a pre-synaptic tracer, and *o*_1_(*t*) and *o*_2_(*t*) represent post-synaptic tracers. Note that each neuron carries its own tracer variable, but the *i* index has been dropped here for clarity. The timescales of the tracer variables were measured in [17] for pyramidal cells in the visual cortex and are as follows: $\tau_{r_{1}}=16.8$ ms, $\tau_{o_{1}}=33.7$ ms, and $\tau_{o_{2}}=114$ ms.

To stabilize network activity, we implement a homeostatic mechanism in the form of a rate detector that acts on a fast timescale, known to stabilize the dynamics induced by the minimal triplet rule into recurrent excitatory networks [18]. This homeostatic mechanism works by allowing the amount of depression, *A*_LTD_(*t*), to change as a function of a moving-average of the post-synaptic firing rate, ${\bar{\mu }}_{E}$:

$$\begin{array}{c} A_{\text{LTD}}\left(t\right)=\frac{\tau_{r_{1}}\;\tau_{o_{2}}\;{\left[{\bar{\mu }}_{E}\left(t\right)\right]}^{2}}{\rho \tau_{o_{1}}}A_{\text{LTP}}, \end{array}$$ (5)

where the timescales $\tau_{r_{1}}$, $\tau_{o_{1}}$ and $\tau_{o_{2}}$, are those from Eq (4), and *ρ* is the target firing rate, chosen to be 8 Hz to replicate the low firing rate of the mouse visual cortex during early development [24]. The moving average of the firing rate, ${\bar{\mu }}_{E}\left(t\right)$, is found by taking a low-pass filter of its spike train as follows

$$\begin{array}{c} {\bar{\mu }}_{E}=\frac{1}{\tau }\sum_{k}\text{exp}(-\frac{t-t_{k}}{\tau }), \end{array}$$

where *t*_*k*_ represents the *k*th spike time that occurred prior to the current time *t*, and *τ* = 1 s. Note that the synaptic strength $\bar{g}\left(t\right)$ in Eqs (2) and (3) can take on either ${\bar{g}}_{\text{LGN}}\left(t\right)$ for synapses from LGN to the cortex, or ${\bar{g}}_{EE}\left(t\right)$ for synapses among excitatory cortical neurons. These synapses have different learning rates, $A_{\text{LTP}}^{\text{LGN}}$ and $A_{\text{LTP}}^{\text{cort}}$, for the LGN feedforward synapses and recurrent cortical synapses, respectively. See Table 1 for a comprehensive list of parameter values used in this work.

**Table 1:** Model parameter values parametrized for the visual cortex. See text for references.

**Neuron Parameters**			
Membrane time constant, *τ*_*m*_	20 ms	Leakage reversal potential, *v*_*l*_	-60 mV
Excitatory reversal potential, *v*_*E*_	0 mV	Inhibitory reversal potential, *v*_*I*_	-80 mV
GJ conductance, *g*_*c*_	0.06	Background firing rate, *ν*	0.5 Hz
Excitatory synaptic time constant, *σ*_*E*_	11 ms	Inhibitory synaptic time constant, *σ*_*I*_	15 ms
background spike strength, ${\bar{g}}_{\text{back}}$	0.02		
**Plasticity Parameters**			
Learning rate for LGN, $A_{\text{LTP}}^{\text{LGN}}$	0.005	LTP time constant, $\tau_{r_{1}}$	16.8 ms
Learning rate for V1, $A_{\text{LTP}}^{\text{cort}}$	0.015	LTD time constant, $\tau_{o_{1}}$	33.7 ms
Triplet LTD time constant, $\tau_{o_{2}}$	114 ms	Maximum LGN weight, $g_{\text{LGN}}^{\text{max}}$	0.02
Maximum I→ E weight, $g_{I}^{\text{max}}$	0.05	Max E→ E weight, $g_{E}^{\text{max}}$	0.025
Learning rate for iSTDP, *A*_iSTDP_	0.008	Time constant, *τ*_iSTDP_	20 ms
Target firing rate, *ρ*	8 Hz		

In addition to the plasticity introduced on the feedforward and recurrent excitatory synapses, we include plasticity on the synapses from inhibitory neurons to excitatory neurons in the cortex [19]. The motivation for including this inhibitory plasticity is that the homeostatic rate detector alone was not sufficient in controlling the firing rate of the network and enabling competition among the synapses. In particular, we found that the stability of the learning process (by this we mean the competition of the weights such that some decay and some grow) was highly sensitive to changes in the learning rate when the homeostatic rate detector was acting alone. With the addition of inhibitory plasticity, we found the system to be significantly more stable for a wider range of parameter choices. Using inhibitory plasticity as a stabilizing mechanism has been done previously [19, 25]. Note that we did not investigate whether inhibitory plasticity alone would have been sufficient to stabilize the dynamics.

The synapse from a pre-synaptic inhibitory cell to a post-synaptic excitatory cell updates according to the rule

$$\begin{array}{c} {\bar{g}}_{EI}\left(t\right)\to {\bar{g}}_{EI}\left(t\right)+\left(x_{E}\left(t\right)-2\rho \tau_{\text{iSTDP}}\right)A_{\text{iSTDP}}\;\;\;\text{if}\;t=t^{\text{PRE}}, \end{array}$$ (6)

$$\begin{array}{c} {\bar{g}}_{EI}\left(t\right)\to {\bar{g}}_{EI}\left(t\right)+x_{I}\left(t\right)A_{\text{iSTDP}}\;\;\text{if}\;t=t^{\text{POST}}, \end{array}$$ (7)

where *A*_iSTDP_ is the learning rate and *ρ* = 8 Hz is the target firing rate of the excitatory cells [the same as in Eq (5)]. Each cell has a tracer variable *x*_*Q*_ for *Q* = {*E*, *I*} that follows the form of Eq (4), where $\tau_{x_{Q}}=20$ ms for *Q* = {*E*, *I*}. Note the interpretation of these plasticity rules: when the spiking of a pre- and post-synaptic inhibitory and excitatory cell, respectively, occurs within a time window of $\tau_{x_{Q}}$, either potentiation or depression occurs at each pre-synaptic (inhibitory) spike [as per Eq (6)], while only potentiation occurs at each post-synaptic (excitatory) spike [as per Eq (7)].

Development is simulated by connecting a subset of the cortical cells by GJs and allowing the LGN synapses onto all excitatory cortical cells to learn for a period of time (which varies in this work), simulating the first postnatal week of development (see S1 Fig). Then, once simulation is in the second postnatal week, gap junctions are turned off [by setting *g*_*c*,*E*_ = 0 in Eq (1)], and recurrent synapses are turned on. Specifically, ${\bar{g}}^{IE}$ and ${\bar{g}}^{II}$ go from zero to nonzero values; ${\bar{g}}^{EE}\left(t\right)$ updates (and LGN synapses continue to update) according to the rules defined in Eqs (2) and (3); and ${\bar{g}}^{EI}\left(t\right)$ updates according to the rules defined in Eqs (6) and (7). We simulate this network for 1200s until the recurrent cortical weights have stabilized and each cortical cell has developed an input preference (called the OP in this work); see S2 Fig for some discussion of the stability of OPs after 1200s. We note that the network operates in an asynchronous regime known to accentuate the performance of STDP [26].

Tuning properties of the cortical cells are determined by taking the final weights from the simulated network and, for each input stimulus preference from 0 to 1000 in increments of 20, we record the firing-rate responses for all neurons averaged over two seconds of simulation time. Tuning curves are calculated for each cortical cell by determining the firing rate of that cell for each input stimulus and normalizing by the maximum firing rate across all cells. The OP of the cortical cell is determined as the stimulus location that gives the greatest response. Selectivity is determined using the orientation-selectivity index (OSI), a measure for selectivity of a cell,

$$\begin{array}{c} \text{OSI}=\frac{R_{\text{pref}}-R_{\text{perp}}}{R_{\text{pref}}+R_{\text{orth}}}, \end{array}$$

where *R*_pref_ is the firing rate of the neuron at its preferred orientation and *R*_orth_ is the firing rate of the neuron at the orthogonal orientation (in this work, the orthogonal orientation corresponds to the orientation that is 500 units away from *R*_pref_). An OSI value close to 1 indicates high selectivity and a value close to 0 indicates no selectivity.

## Results

We describe simulation results for three realizations of the cortical network, each with progressively more realistic connectivity properties. These realizations of the network are chosen to demonstrate three characteristics of synaptic development in the presence of GJs: (i) The development of feedforward LGN synapses onto cortical cells in the the presence of GJs; (ii) The effect of GJ-coupling on the formation of cortical all-to-all synapses; (iii) The development of an OP map when cortical synapses are spatially restricted.

### GJs and receptive field development

We begin by studying the development of the feedforward LGN synapses onto the cortical cells. Specifically, we use a 400-neuron cortical network in which 20% of the cells are inhibitory and 80% are excitatory. We allow two excitatory cells to be coupled by a GJ with a 50% probability such that about half of the excitatory population is GJ-coupled in pairs (similarly to the small proof-of-concept model network explored in [21]). Following the experimental timeline (see S1 Fig), we simulate the first postnatal week of development by allowing the feedforward synapses to learn, via the rules discussed in the Methods and Models section, for 600 seconds of simulation time. During this time, excitatory cells are GJ-coupled while the recurrent synaptic connections are set to zero (phase 1 of development). At the end of this phase, we turn off the GJs between cortical cell pairs and allow recurrent cortical synapses to learn together with the feedforward synapses from the LGN (phase 2 of development).

**Fig 2:**
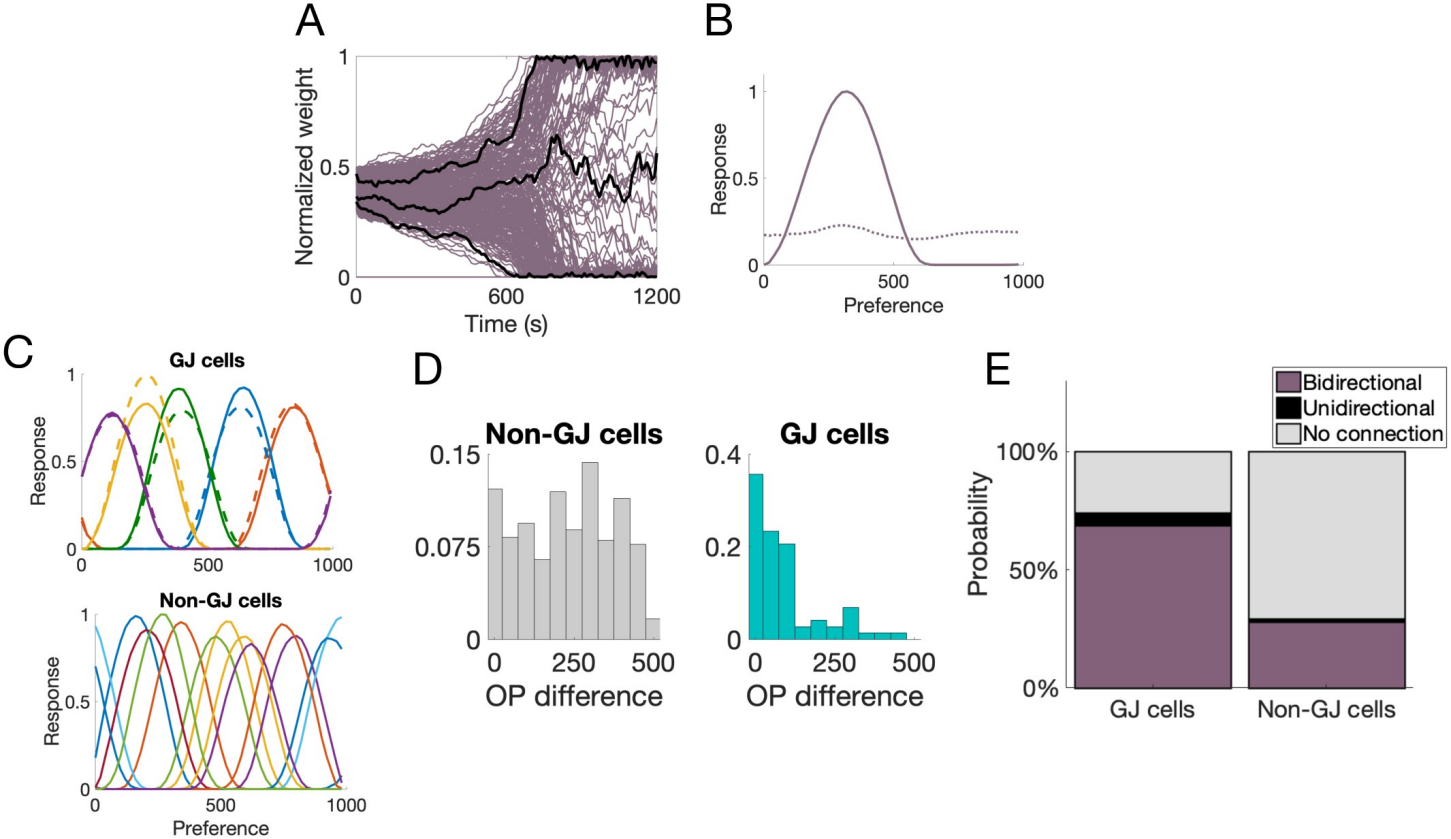
Measurements from a 400-neuron network with pairwise GJ coupling. **A**: Progression of the synaptic weights from LGN to one sample excitatory cortical cell. The black curves highlight three synapses that have potentiated, depressed, and remained around an average value; **B**: Tuning curve of this sample cell before (dotted curve) and after (solid curve) feedforward LGN synaptic learning. Recall that OP is labeled according to LGN input labels 0–1000; **C**: Sample tuning curves. (top) Tuning curves of five GJ-coupled pairs, where matching colors indicate the GJ-coupled pairs. (bottom) tuning curves of non-GJ-coupled excitatory cells; **D**: Distribution of the difference in OP between GJ-coupled pairs (teal) and non-GJ-coupled cells (gray); **E**: Probability of a bidirectional synapse (purple), a unidirectional synapse (black), or no synapse at all (gray) between GJ-coupled cells and non-GJ-coupled cells.

Due to the competitiveness of the STDP learning rule, about half of the LGN synapses onto one cortical cell potentiate to the maximum possible synaptic strength and half are depressed to zero, see Fig 2A. Further, the synapses that become potentiated tend to have a similar labeling (i.e., respond preferentially to a similar input value), resulting in an input preference for the cortical cell at the end of the simulation, see Fig 2B. This input preference is what we refer to as the OP of the cortical cell in this work. The cells that were coupled by a GJ during the first phase of development, i.e., during the time of feedforward learning, develop similar OPs, while cells that did not contain GJ coupling are not likely to share an orientation preference, see Fig 2C and 2D. Finally, the model reproduces the experimentally-observed behavior for GJ-coupled cells to preferentially form bidirectional synapses, see Fig 2E. Note that the probability of finding bidirectional synapses between GJ-coupled cells is much higher in the model than those observed in real cortex (26% in [7] compared to almost 80% here) since we are directly comparing GJ-coupled cells, while the experiments tested all sister cells (only a fraction of which are coupled by a GJ).

**Fig 3:**
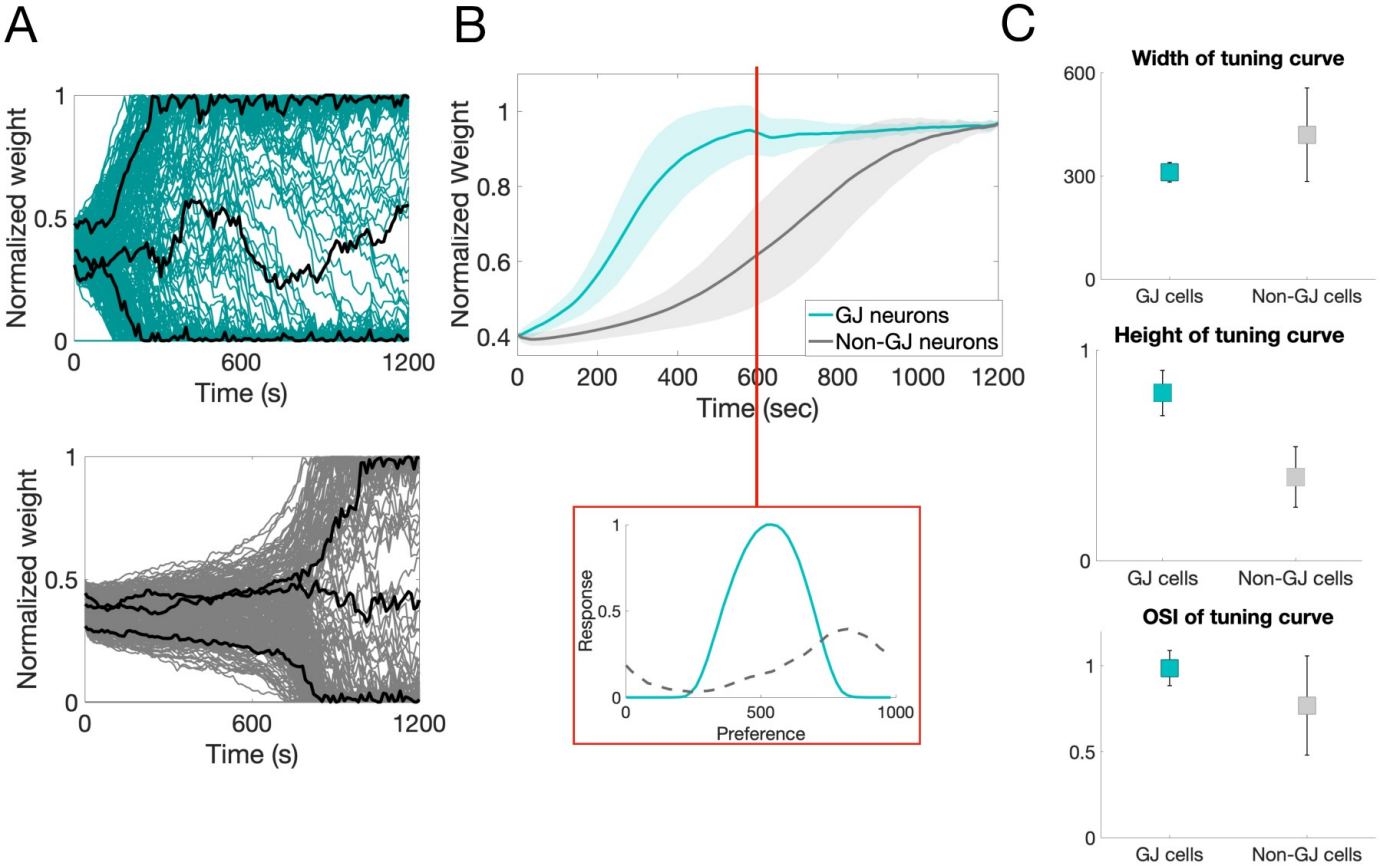
Rate of learning properties of GJ- vs. non-GJ-coupled cells. **A**: Top (bottom): Progression of the feedforward synaptic weights onto a sample GJ-coupled (non-GJ-coupled) neuron. The black curves highlight three synapses that have potentiated, depressed, and remained around an average value; **B**: The average weight progression (curve) and standard deviation (shaded region) for all GJ-coupled neurons (teal) and all non-GJ-coupled neurons (gray) calculated by averaging together all feedforward synapses that potentiated to at least 70% of the maximum synaptic weight over all cells in each population. Inset: Sample tuning curve for a GJ-coupled neuron (solid) and a non-GJ-coupled neuron (dashed) after 600 seconds of simulation time, before recurrent connections begin to form. **C**: Width and height of the tuning curves measured for all cells in the network, as well as the orientation-selectivity index (OSI), the average reported as the center of each square, the standard deviation as error bars, over all cells in each group.

Our simulations show that GJ-coupled cells tend to develop an OP much sooner than non-GJ-coupled cells. The feedforward synapses from LGN onto the GJ-coupled cells learn much faster than those synapses onto cells that are not GJ-coupled, see Fig 3A. This effect is consistent across all GJ-coupled and non-GJ-coupled pairs; Fig 3B. Notice that the slope of the average synaptic strength is much larger for those cells with GJ coupling during the first phase of development than for those cells without.

Due to this increased learning rate, GJ-coupled cells are more selective for orientation (have more clearly-defined tuning curves) than non-GJ-coupled cells. To demonstrate this, we measure properties of the tuning curves of the GJ-coupled and non-GJ-coupled neurons at the end of the first phase of development, before cortical synapses learn. Fig 3B (inset) shows the tuning curve of a GJ-coupled cell (teal) and non-GJ-coupled cell (gray) at the end of the first phase of development. Notice that the GJ-coupled cell has more selectivity than the non-GJ-coupled cell, as indicated by the tall thin peak. This effect is quantified over all cells in the network by considering the width and height of the tuning curve for all GJ-coupled cells and non-GJ-coupled cells. Fig 3C shows the average over all GJ-coupled (teal) and non-GJ-coupled (gray) neurons for three measures of orientation selectivity. Notice that GJ-coupled neurons clearly have more selectivity than non-GJ-coupled cells at the time that cortical synapses begin to form.

### Recurrent synapse formation (all-to-all network)

Next, we study the effect of GJ coupling on the formation of cortical synapses. We make the model more realistic by including sister-cell groups in the excitatory population and coupling a percentage of them with GJs (rather than simple pair-wise coupling as in the previous section). We also explore varying the time at which the synapses between cortical cells form to investigate how GJ coupling during the first developmental phase affects the resulting OPs of the cortical cells. We also decrease the model network size from 400 to 256 neurons to speed up computation time while ensuring that there are still enough excitatory cells (∼ 200) to measure GJ properties when the coupling is sparse.

**Fig 4:**
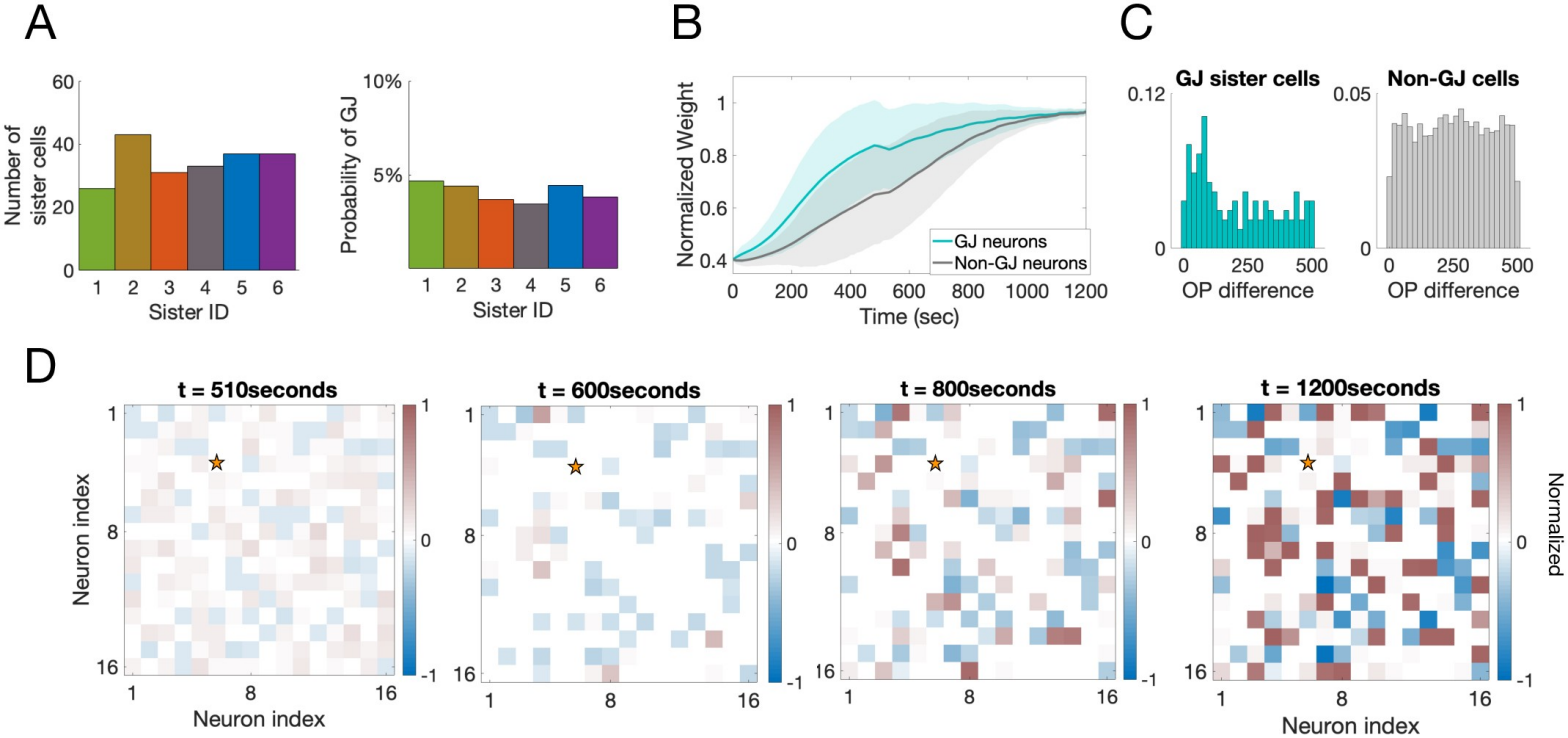
Measurements from a 256-neuron network with all-to-all potential cortical connectivity. **A**: The number of cells (left) and the probability GJ-coupling (right) within each of the six sister groups. **B**: The rate of LGN synaptic learning averaged over all GJ-coupled cells (teal) and non-GJ-coupled cells (gray). **C**: The distribution of differences in OP for GJ-coupled cells (teal) and non-GJ-coupled cells (gray). **D**: The normalized recurrent cortical weights onto one sample excitatory cell, indicated by a star. Inhibitory weights onto this cell are indicated by negative values and shown in blue while excitatory ones are positive and shown in red. The recurrent connections begin at *t* = 500 seconds and the entire simulation was run for 1200 seconds.

To create sister-cell groups, we divide the excitatory population into six groups with equal probability, where each group represents a set of sister cells (i.e., all cells in each group are sister cells to only those cells in that group). The motivation behind choosing six groups of sister cells is that, in mouse V1, sister cells are intermingled with other sister cells and outnumbered in a local volume by a factor of six [27]. We assume that 256 neurons corresponds to a small enough volume of the cortex that we can consider only six groups of sister cells that are randomly distributed in the space. Within each sister-cell group, each neuron has a 5% probability of being coupled to a sister cell by a GJ. Fig 4A shows a count of the number of cells in each sister group along with the probability of GJ coupling in each group. Note that this coupling percentage is much sparser than the ∼ 28% coupling probability measured experimentally for radially-aligned sister cells [7]. We found that is was necessary to require a sparse GJ-coupling during the first postnatal week for the GJ-coupled cells to exhibit the experimentally-measured properties of OP sharing and preferential synaptic coupling. We will discuss this more in a later section.

The response properties measured for the pairwise GJ-coupled 400-neuron network remain in this 256-neuron network, including the increased rate of learning for GJ-coupled cells compared to non-GJ-coupled cells, see Fig 4B, and the preference for GJ-coupled cells to share an OP, see Fig 4C. The recurrent synapses between excitatory cells can be all-to-all, as illustrated in Fig 4D by the red boxes being scattered throughout the entire cortical region, but due to the competitive STDP rules, each excitatory cell forms a strong synapse with only about half of the other excitatory cells (the other half decay just as in the feedforward LGN synapses).

**Fig 5:**
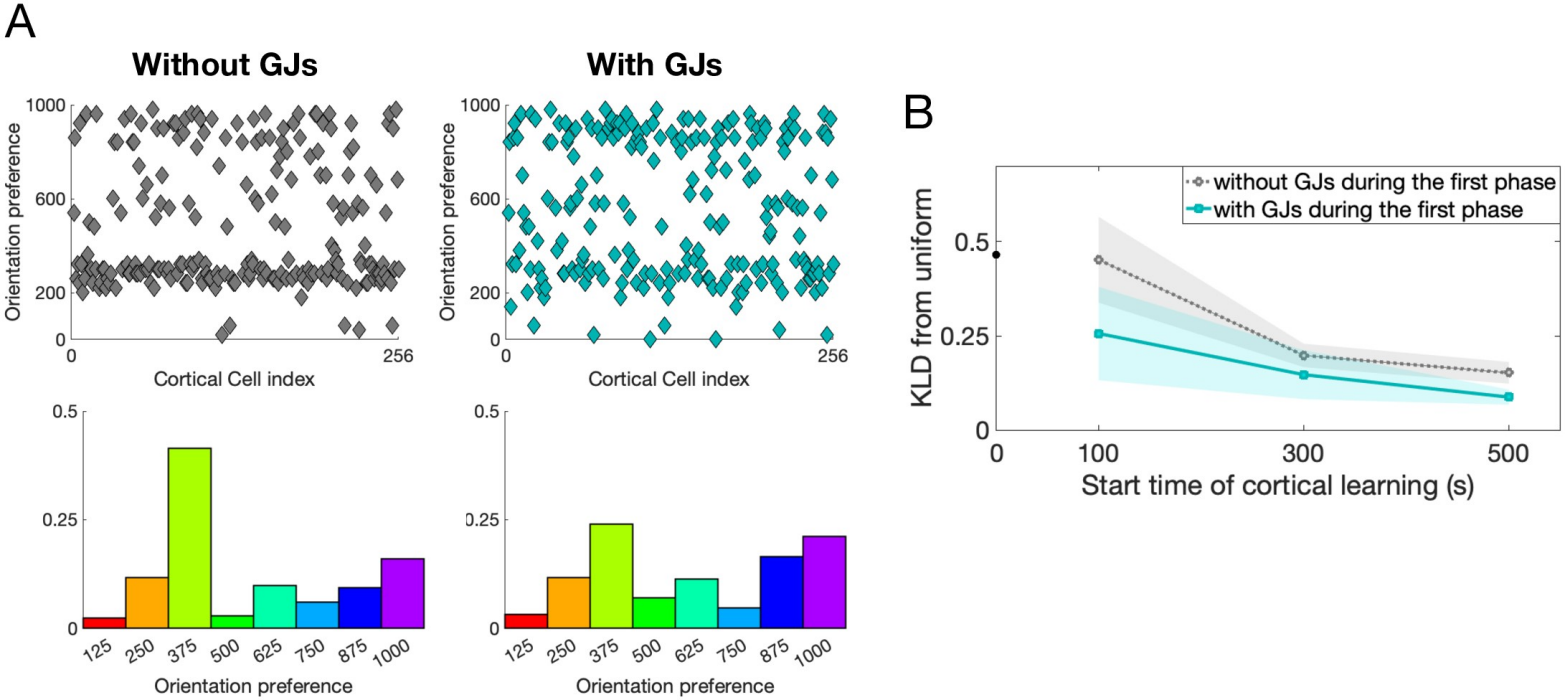
Distribution of OPs comparing a network containing GJ coupling to one without. **A**: (Top) Plot of each cell’s OP; (Bottom) distribution of OPs, for a network that does not contain (left) and one that does contain GJ coupling (right) during the first phase of development. The start time of cortical learning is 100 seconds. **B**: The average Kullback-Leibler divergence (KLD) between the uniform distribution and the OP distribution for the networks with (solid teal) and without (dotted gray) GJ coupling during the first phase. The curves are the average, and the shaded region the standard deviation, across 5 trials. Smaller values indicate distributions more similar to the uniform distribution.

We now begin to investigate how these effects from GJ coupling during the first phase might affect the OP of each cortical cell. First, we show that if GJ coupling is turned off during the time that feedforward LGN synapses are learning (the first phase of development), the distribution of OPs that forms has more order than the one that forms when GJs are present during the first phase of development. In particular, when cortical recurrent connections form between all cells in the network, all cortical cells develop a similar OP (as seen and discussed in [16]). Fig 5A shows that the OPs in the network without GJ coupling tend to cluster around one value (∼ 375), indicating that the recurrent connections influence the resulting OP of each cell, while the network with GJ coupling during the first phase of learning has a more uniform distribution of OPs. To quantify this, we calculate the Kullback-Leibler divergence (KLD) between each resulting OP distribution and the uniform distribution; see Fig 5B. Notice that networks without GJ coupling during the first phase of development have a lower KLD value than networks containing GJ coupling, indicating that the inclusion of GJ coupling during the first phase of development results in an OP distribution that is more similar to the uniform distribution, where each OP has equal likelihood of occurring.

In addition to GJ coupling, the time at which recurrent synapses begin to learn (the start time of the second phase of development) also has an effect on the distribution of OPs. Specifically, the amount of disorder (closeness to a uniform distribution) increases with the start time of recurrent synaptic learning as shown in Fig 5B. Next, we apply this idea to a cortical network with spatially-restricted synaptic connectivity.

### Recurrent synapse formation (radius of cortical connectivity)

Next, we introduce spatial restrictions on the cortical synaptic connectivity and compare the resulting OP maps across networks that contain GJ-coupling during the first phase of learning and networks that do not. To introduce spatial effects into the model, we draw a fixed radius around each excitatory cortical cell and only allow excitatory synaptic connections from cells within that radius. Note that excitatory to inhibitory, inhibitory to excitatory, and inhibitory to inhibitory synaptic connections still remain all-to-all, with no spatial restrictions. The excitatory to excitatory synaptic strengths are plastic, following the triplet learning rule, while the inhibitory to excitatory synapses are also plastic, following the iSTDP learning rules as described in the Methods and Models section.

**Fig 6:**
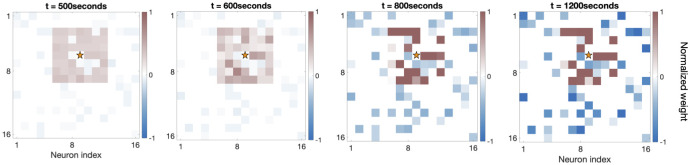
Evolution of the strength of the recurrent synapses onto one sample excitatory cortical cell shown at different time points during the second phase of development. The star indicates the location of the sample neuron. Inhibitory weights onto this cell are indicated by negative values and shown in blue while excitatory ones are positive and shown in red.

Fig 6 shows the development of recurrent synapses onto one sample excitatory neuron in the network. In this example, excitatory recurrent connections within a radius of 4 units are turned on at time t = 500 seconds, with initial weights chosen randomly from the interval [0.25, 0.35]*g*_*max*_. As cortical synaptic learning progresses, about half of these excitatory recurrent synapses (within the radius) are potentiated, while half are depressed, as expected and shown in previous sections. Notice that negative weights indicate inhibitory synapses onto this excitatory example neuron, which potentiate as the excitatory weights increase to mediate the firing rate of this cell.

**Fig 7:**
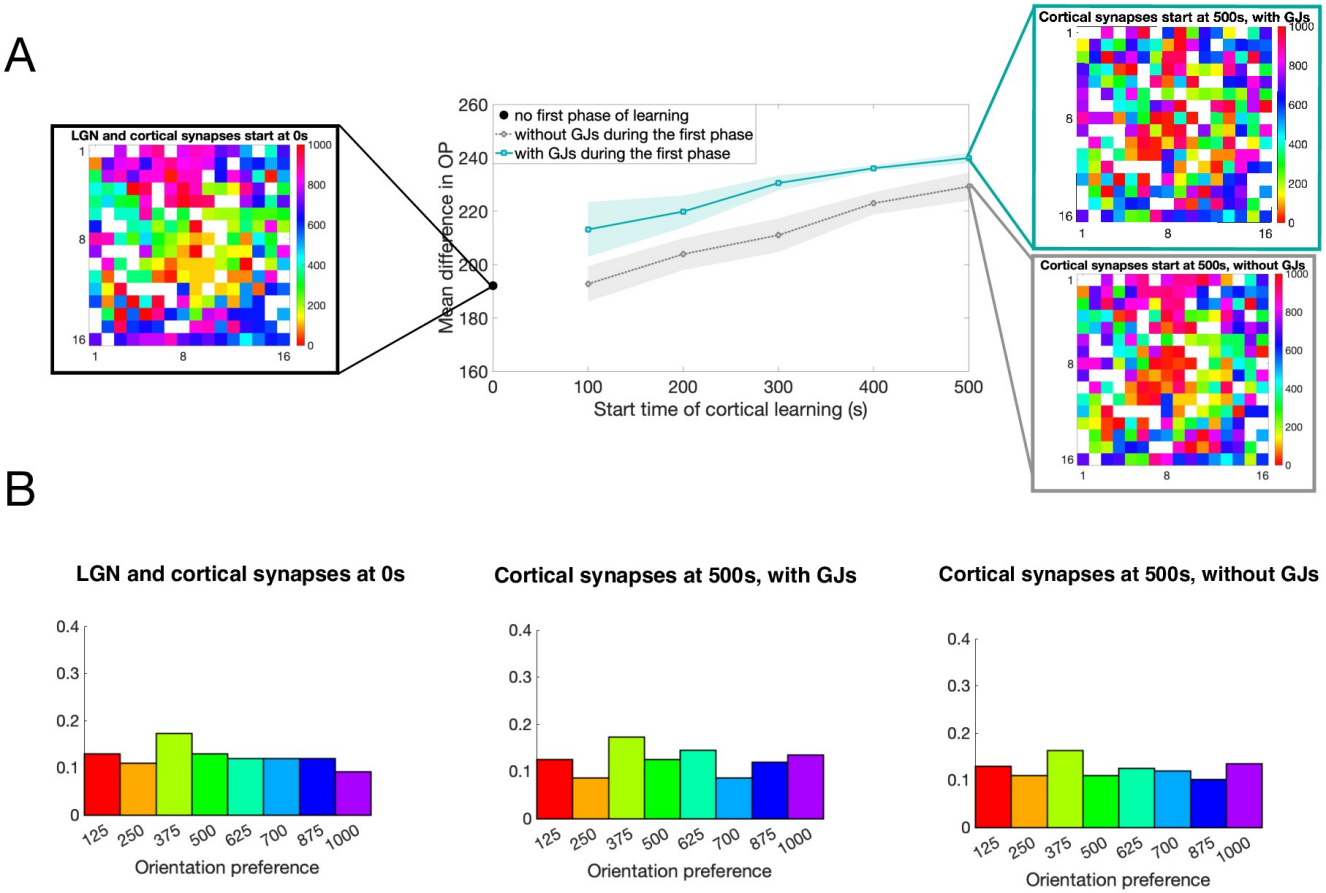
Effect of GJ-coupling and timing of cortical learning on the OP. **A**: The plots shown are OP maps for different types of networks where the color indicates the preference of the cell at that location and the white boxes indicate the inhibitory cells (that do not have a preference). The leftmost map is for a network in which the recurrent synapses form at the same time as the feedforward LGN synapses, the top right map is for a network that contains GJ-coupling during the time that feedforward LGN synapses are forming, and the bottom right map is for a network that does not. The graph in the middle shows the average difference in OP (as defined in the text) for cells within a radius of 4 units, where higher values indicate disorder. The curves are the average, and the shaded region the standard deviation, across 5 trials. The horizontal axis denotes the time at which recurrent synapses within the cortex begin to learn (start time of the second phase of development). After this time, if there were GJs in the network, they are turned off. Note that, for the case of cortical synapses beginning at 0s, there are no GJs in the network by definition since there is no first phase of development. **B**: Distribution of OPs for the three networks in **A**.

We investigate the effect of GJ-coupling during the first phase of development by measuring the amount of order in the resulting OP map with and without GJ coupling during this phase; see Fig 7A. The leftmost plot shows the OP map for a network in which the recurrent synapses form at the same time as the LGN synapses. Notice that there are patches of cells with similar OPs, the sizes of which correspond to the radius of connectivity. If we increase the amount of time that the LGN synapses change without recurrent cortical synapses to 500 seconds (the first phase of development), we observe that the degree of disorder increases until we reach a salt-and-pepper map, see rightmost plots of Fig 7A, with the network containing GJ coupling during the first phase of development (top) exhibiting a higher degree of disorder than the network that did not contain GJ coupling during the first phase (bottom).

We quantify the degree of disorder in the OP map by calculating the average difference in OP for each cell within the radius of cortical connectivity. For each excitatory cell, we take the difference between the OP of that cell and the OP of the excitatory cells that are within the radius of connectivity (4 units) and then take the average of those differences. Finally, we take the average of this OP difference over all of the excitatory cells in the network to obtain the measure shown in Fig 7A. Notice that larger values of this measure indicate larger differences in OP, which corresponds to more disorder. We observe that the degree of disorder in the OP map increases as the start time of the cortical synapses (the length of the first phase of development) increases. The networks in which GJ coupling is present during the first phase of development follow this same trend as the start time of cortical synapses increases, but also exhibit overall higher levels of disorder than those networks that did not contain GJ coupling; see solid teal curve as compared to dotted gray curve in Fig 7A.

We observe that, in these last two realizations of the network model (all-to-all connectivity and radius connectivity), the overall OP distribution is close to uniform; see Fig 5A and 5B. Though each orientation has about equal representation in all example networks, the spatial distribution of the cells with each OP changes drastically across each network depending on GJ-coupling and the timing of recurrent synapses.

### Effect of GJ-coupling density on synchrony

The mechanism underlying the shared OP of GJ-coupled cells is the synchrony (or strongly correlated spike times) induced between the two cells by the GJ. As the feedforward LGN synapses form, cells that fire synchronously preferentially develop a similar set of strengthened LGN synapses, and thus form a similar OP. In our model, sparsity of GJ coupling between the sister cells is essential for this synchrony to occur, and consequently, for the shared OP of GJ-coupled cells.

**Fig 8:**
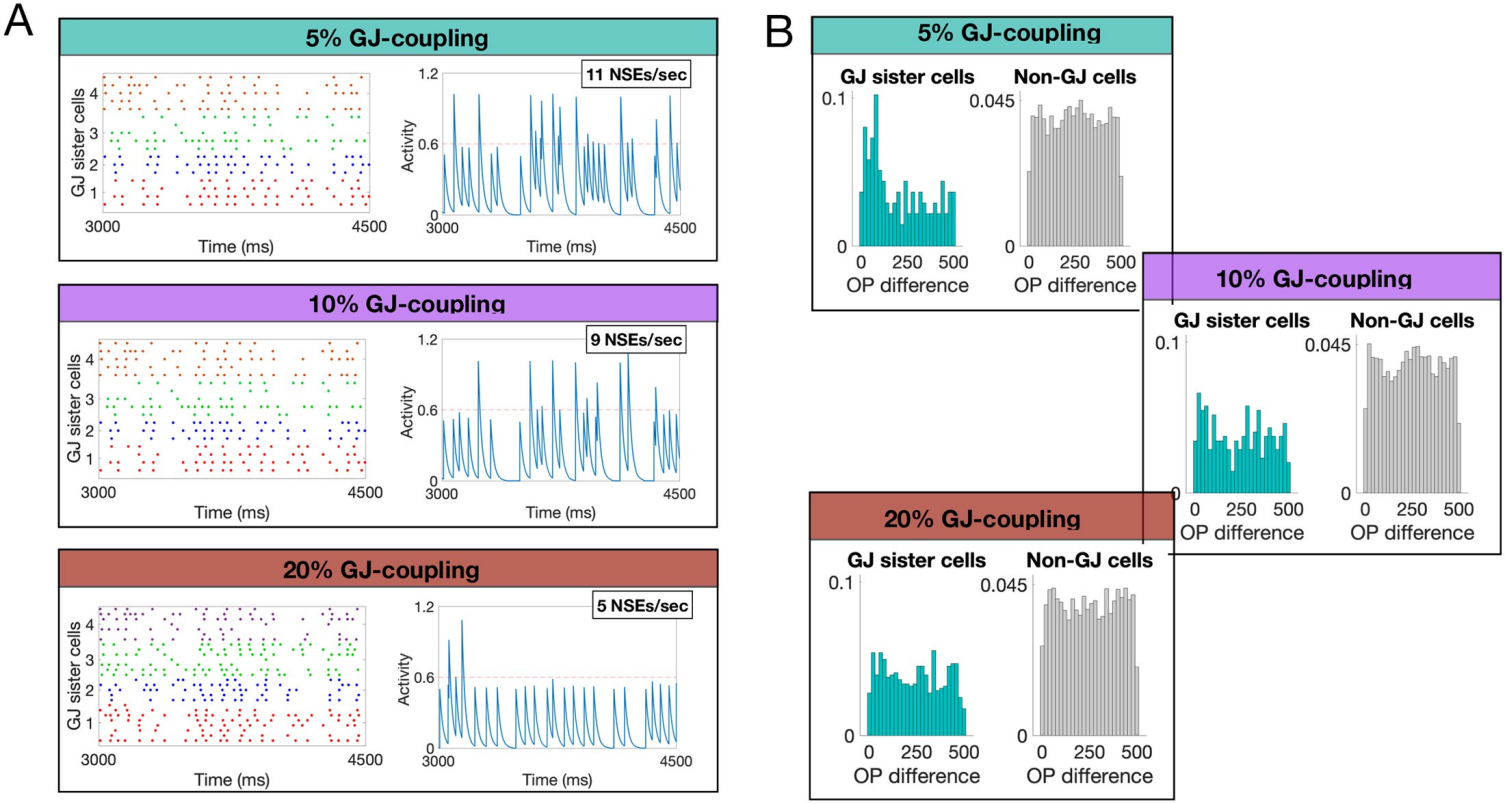
**A**: Sample raster plots (left) and average activity (right) of sets of GJ-coupled sister cells for different GJ coupling percentages. Each color-coded set of spike times in the raster plots indicate a set of GJ-coupled sister cells. The activity plots shown to the right corresponds to the GJ-coupled set shown in red in each raster plot. The average activity plots were created by adding an exponential tail of 20 ms to each spike for each cell in the GJ group, adding them together, and dividing by the number of cells in each group. The average number of network synchronous events (NSEs) per second, a measure for the amount of synchrony, for each coupling percentage, is shown in text on the average voltage plots in **A**. The threshold for determining an NSE was chosen as 0.6, as illustrated by the red dashed line, though small changes in this threshold do not significantly affect the results. **B**: The difference in OP for GJ-coupled cells (teal) and non-GJ-coupled cells (grey) in a network with 5%, 10%, and 20% GJ coupling among sister cells (top to bottom).

First, we show that synchrony decreases as a function of the percentage of GJ-coupling among sister cells; see Fig 8A, which shows sample raster plots and average activity plots for GJ-coupled sister cells with different coupling percentages. The intuition for this dependence of synchrony upon density is as follows: When cells are coupled with a probability of 5%, each cell is coupled to an average of 1.5 other cells, leading to isolated pairs or triplets of GJ-coupled cells whose only communication is with their GJ-coupled partners. As the coupling percentage increases, the GJ-coupled cells are no longer isolated; rather, each cell may be coupled to several different groups of GJ-coupled sister cells, leading to an overall desynchronization.

To determine the effect across many GJ-coupled sets of sister cells, we include a measure for synchrony that involves counting the number of times the network crosses a threshold (chosen as 0.6 in this work), deemed a Network Synchronous Event (NSE) as has been done in previous work [4, 5]. In this measure, higher values indicate that more GJ-coupled cells are firing within a short timeframe and thus are more synchronized. As was illustrated in the raster and average activity plots, the amount of synchrony between GJ-coupled cells decreases with an increase in coupling percentage. A direct consequence of this desynchronization with coupling percentage is that GJ-coupled cells no longer share an OP after the feedforward synapses have stabilized; see Fig 8B.

**Fig 9:**
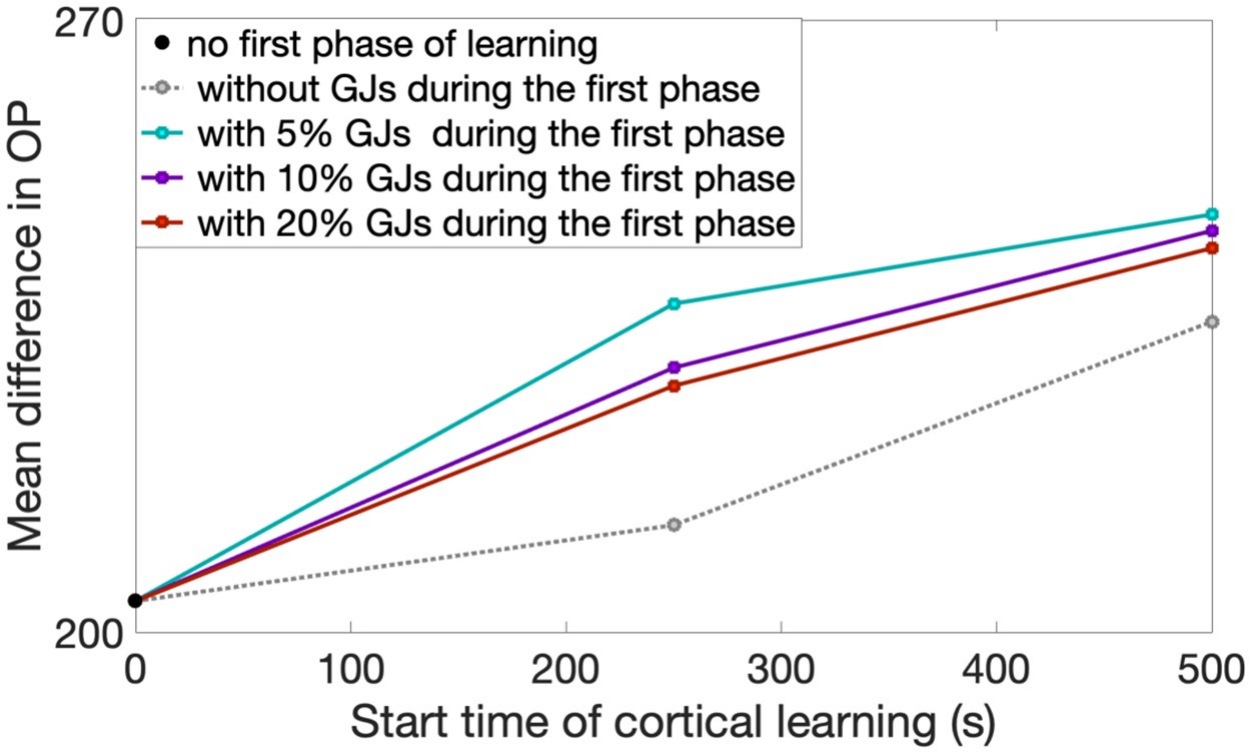
The mean difference in OP for varying start times of the cortical synaptic learning comparing different percentages of GJ-coupling between sister cells. Note that we only show results for greater than 250 seconds of cortical learning to ensure the GJ-coupling has time to affect the dynamics.

Though the preference for GJ-coupled cells to share an OP is diminished with increasing GJ-coupling probability, all other properties of OP-map development, such as the discussion of order vs. disorder, do not rely on this assumption; see Fig 9. In addition, though experiments measure the GJ-coupling percentage between sister cells during the first postnatal week as about 28%, this was specifically measured for isolated pairs of radially-aligned sister cells [7]. In this work, we are interested in sister cells that are GJ-coupled laterally (within the layer), which hasn’t explicitly been measured.

## Discussion

We have created a simple mathematical model to further understand how GJ coupling among sister cells early in development might affect the formation of the receptive fields of V1 cells, as well as seed the cortical maps that develop later. The model uses spike-timing dependent plasticity (STDP) rules explicitly parametrized for the visual cortex to explore potential mechanisms underlying the formation of ordered or disordered orientation preference (OP) maps. Predictions from our model include a faster rate of learning for GJ-coupled cells than for non GJ-coupled cells; an increase in disorder in the OP maps for networks with GJ-coupling (dependent on sparsity of such coupling); and a relationship between the relative timing of plasticity of the feedforward LGN synapses and recurrent cortical synapses.

There is a vast literature on the role of Hebbian plasticity in forming circuits and OP map development, see for example [28–32] and [33–37]. Our model aligns most closely with the work in [16, 17, 38], with the inclusion of GJs as in [21], but with a focus on the effect of GJ coupling during development on OP map formation. When compared with previous work on the effects of GJs on development of the visual cortex, our model includes a larger network, more realistic plasticity rules for the visual cortex, and two phases of development (plasticity of feedforward LGN synapses before recurrent synapses and plasticity of recurrent synapses together with feedforward LGN synapses) to propose a mechanism underlying disordered OP maps in mice.

**Fig 10:**
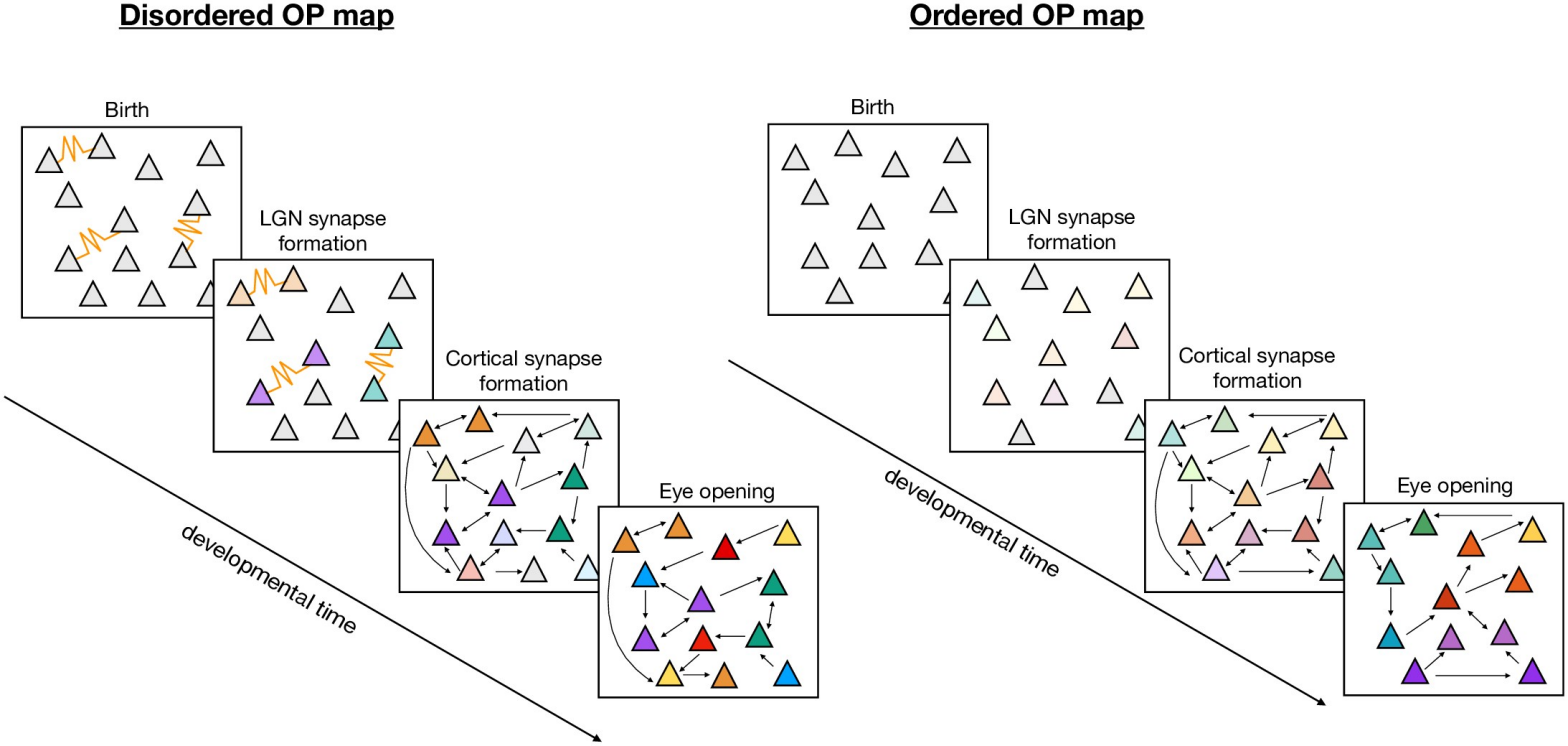
A schematic representing how GJs during the first week might lead to a disordered (i.e., salt-and-pepper) OP map. Each stage drawn here represents: (i) The first postnatal week as labeled by “Birth” and “LGN synapse formation”, (ii) the second postnatal week while chemical synapses are forming, and (iii) the resulting cortical recurrent synapses and OP as indicated by the color of the cell. Transparency represents selectivity, where opaque colors indicate a higher amount of selectivity or sharp tuning.

Specifically, our model shows that GJ-coupled cells exhibit higher firing rates and faster rates of learning for their feedforward LGN synapses than their unconnected counterparts, leading to higher selectivity of GJ-coupled cells at the time recurrent synapses begin to form, a result that has not, to our knowledge, been shown before. The implication of GJ-coupled cells having more selectivity than non-GJ-coupled cells is that their tuning properties are less likely to be influenced or changed by the recurrent cortical synapses when they begin to form during the second phase of development. If we consider sparsely-coupled GJs during the first phase of development, and expect that GJ-coupled cells will preferentially develop similar OPs while different sets of GJ-coupled cells develop different OPs, then we expect to see pairs of cells with similar OPs scattered throughout the cortex. Assuming that the GJ-coupled cells are sufficiently selective at the initiation of recurrent cortical learning such that their OP does not change during this second phase, one would expect that the final OP map in this case would be salt-and-pepper, as demonstrated in the left schematic of Fig 10. On the other hand, if GJs did not exist during the first phase of development while LGN synapses were forming, then the cortical cells would not be sufficiently selective by the time that recurrent synapses formed, and the development of the cortical recurrent synaptic connections would influence the final OP of each cell. This might result in an OP map that has order, as demonstrated in the right schematic of Fig 10. We have shown in Fig 7 that indeed the inclusion of GJs during the first phase of development leads to maps that are more disordered than their non-GJ-coupled counterparts.

We also explore the effect of timing of the recurrent cortical synapses on OP map development. Specifically, we show that order in the OP map increases with earlier formation of recurrent synapses, independent of the existence of GJs during the first phase of learning. This leads us to conclude that GJ coupling during the first phase of development indeed promotes a disordered OP map, but works together with the relative timing of synaptic development from LGN and within the cortex.

In our work, synchrony of the spike times between GJ-coupled sister cells underlies the formation of similar OPs for those coupled cells, while the sparsity of GJ coupling among sister cells underlies the enhancement of disorder in the OP map. In particular, we show that as the density of GJs among sister cells increases, synchrony decreases, leading to a decrease in the preference for coupled cells to share an OP. The sparse coupling among sister cells is an assumption of the model, but is reflected in experiments. In particular, sister cells are derived from radial glial cells and migrate to their end location by traveling down the axon of the glial cell. While traveling, these sister cells begin dispersing laterally such that by the end of the second postnatal week, they are dispersed up to 500 *μ*m in radius [13]. Then, sister cells become sparsely intermingled in the mouse visual cortex, with sister cells outnumbered by non-sister cells in a local volume (100–500 *μ*m in diameter) by a factor of six [39, 40], a property that seems to be essential for proper synaptic development [41]. Currently, GJs have only been measured between nearby radially-aligned sister cells (within a radius of 100–120 *μ*m [8]) and the coupling percentage was found to be about 28% for cells distributed throughout several layers [7]. Therefore, it is not unreasonable to assume that sister cells are sparsely coupled laterally at about 5%, though more experiments would be necessary to justify this assumption.

We note also that the model developed in this work is highly simplified, especially in its size, spatial structure, and LGN input organization. In particular, our model lacks non-visually stimulated neurons, and so its idealized LGN feedforward input doesn’t reflect changes in network stimulation after eye-opening (i.e., spontaneous activity to visually-driven input). As the goal of our study was to understand the formation of OP maps due to GJ connectivity during the first postnatal week, we did not address the refinement of recurrent synapses after eye-opening that is observed in [21] and modeled in [38]. Another limitation of the model is the simplicity of the patches of similar OP in our model. Ideally, the ordered map would include the pinwheels of OP observed in the cortices of cats and ferrets, but the formation of ordered pinwheels would require a much larger network than the current model (on the order of thousands of neurons [36]). In this work, network size does not affect the mechanism of disorder (neither does the radius used to measure disorder; see S3 Fig); rather, the density of GJ-coupling among sister cells is one main contributor to the observed dynamics.

Experiments show that visual input is not necessary for cortical cells to develop an OP [42, 43]. Instead, spontaneous activity in the cortex is generated from intra-cortical circuits, as well as input from spontaneous retinal waves [44], and drives synaptic plasticity during the first two postnatal weeks [21]. By the end of the second postnatal week, a weak OP map has already developed and then becomes further stabilized by visual input through the newly-opened eyes. Our model predicts that GJs between sister cells during the first postnatal week produce synchronous coupling between pairs of cells that seeds the functional selectivity that forms later in development, one consequence of which is a disordered “salt-and-pepper” OP map. At eye-opening, experimental data [21] does not confirm the presence of functional selectivity. Rather, at best, there may be only a weak functional selectivity at eye-opening—more prevalent when measured with natural images than oriented gratings—with this functional selectivity increasing significantly over the following postnatal week as visual input is received through the eyes [21]. In the first part of the second postnatal week, very shortly after eye-opening, other experimental measurements [7, 8] show a significant presence of cells, previously GJ-connected sister cells, preferentially sharing OPs and being synaptically coupled. Our model has a higher level of functional selectivity at eye-opening than observed in the data of [21], but the model’s level is consistent with receptive fields developing prior to recurrent synaptic plasticity and with GJs aiding in the development of those receptive fields.

To summarize, our results are consistent with the assertion that GJs during the first postnatal week seed functional selectivity, leading to disordered OP maps. Specifically, our model reproduces the preference for cells that were GJ coupled in the first phase of development to share an OP and preferentially develop a bidirectional synapse later in development, and goes further to suggest how the observed disordered OP map may develop with GJ-coupling between sister cells early in development. The model reproduces experimentally-measured properties of GJ-coupled cells and uses these properties to propose two mechanisms affecting the formation of salt-and-pepper OP maps in the mouse V1: the presence of GJs during the first postnatal week and the relative timing of cortical synapse formation to the timing of feedforward LGN synapse formation. Additional computational studies of networks including realistic LGN input and spatial organization of the cortex, together with the inclusion of plastic GJs between FS inhibitory cells [14] during the second phase of development, are necessary to further extend our understanding of potential roles for GJs in V1 during development.

## Supporting information

S1 Fig Schematic of plasticity and connectivity timeline. **A**: Illustration of the plasticity rules used in this work. **B**: Timeline of biological connectivity of mouse V1 and schematic of model connectivity for the first three postnatal weeks.
(TIF)

S2 Fig Orientation preference has stabilized by 1200s of simulation time. **A**: Plot of the change in feedforward weights to two sample cortical cells, one with GJs during the first phase of development (left, teal) and one without (right, gray), for 3000s of simulated time. The black curves highlight one weight that increased and one that decreased. Notice that by 1200s, we see a clear split of the weights and this cell has developed an OP. **B**: The resulting OP map for a simulation run for 1200s (left) and 3000s (middle) together with the difference in the OP between the two (right). The white squares indicate inhibitory neurons, which are not selective. The mostly-red plot shows that the OP for each neuron does not change much with longer simulation time.
(TIF)

S3 Fig The effect of changing the measurement radius on the average difference in OP. The panels show different start times of the recurrent cortical synapses increasing from left to right. The solid colored lines in each panel indicate those networks that contain GJ coupling during the first phase of development, while the dotted lines indicate those networks that do not. The black curve is for the case when cortical recurrent synapses and LGN feedforward synapses begin at the same time (independent of GJs). Notice that the measure is low (there is order in the OP map) for small radii, and increases with increasing radius, implying that cells share an OP at small distances, but not at large distances. Importantly, for the networks containing GJ coupling during the first phase of development, and for cases in which the feedforward synapses were allowed to learn for a sufficient amount of time while the GJs are present (300s and 500s), there seems to be little order for any value of the radius, see blue and orange solid lines in the first and third panels. For the same amount of feedforward learning, there is significantly more order in networks that did not contain GJ coupling, see dotted curves in all panels.
(TIF)
