# Supplementary figures and images for: Modeling the role of gap junctions between excitatory neurons in the developing visual cortex
Source: PLoS Comput Biol. 2021 Jul 6;17(7):e1007915. doi: 10.1371/journal.pcbi.1007915 (PMC8284639; doi:10.1371/journal.pcbi.1007915)

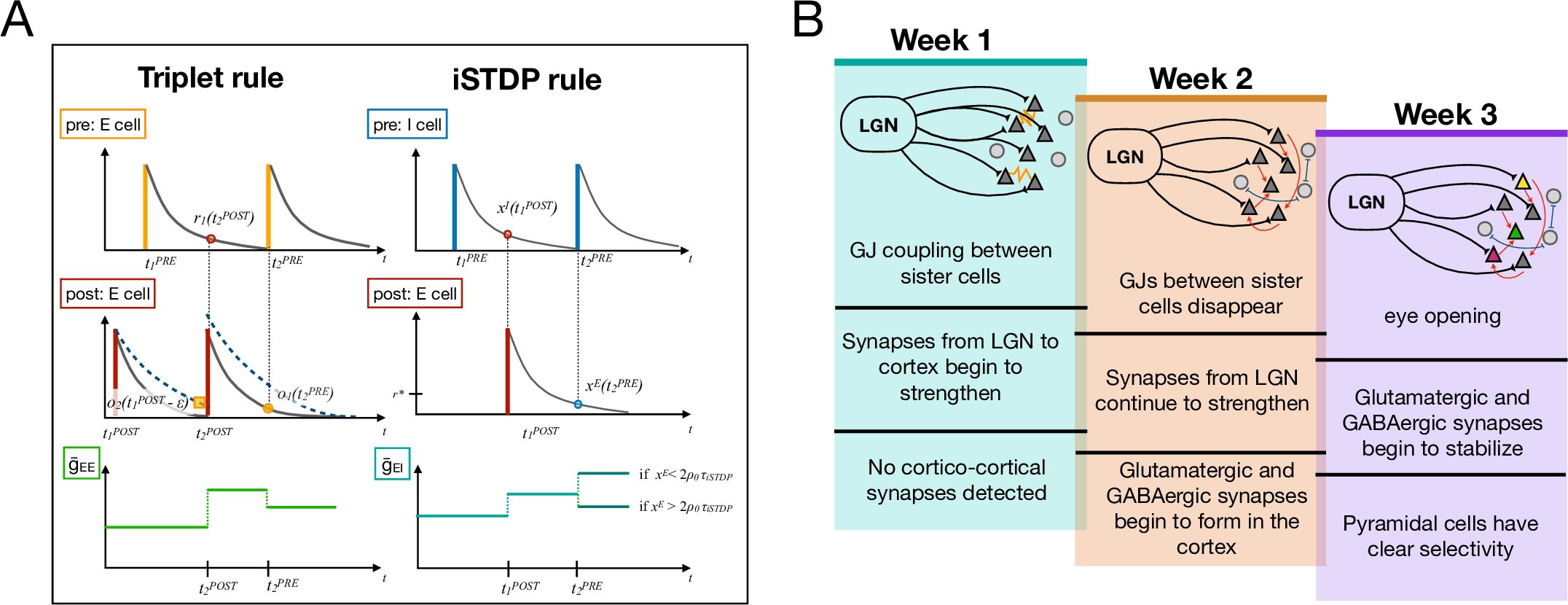

Supplement: S1 Fig — A: Illustration of the plasticity rules used in this work. B: Timeline of biological connectivity of mouse V1 and schematic of model connectivity for the first three postnatal weeks. (TIF) [file pcbi.1007915.s001.tif]

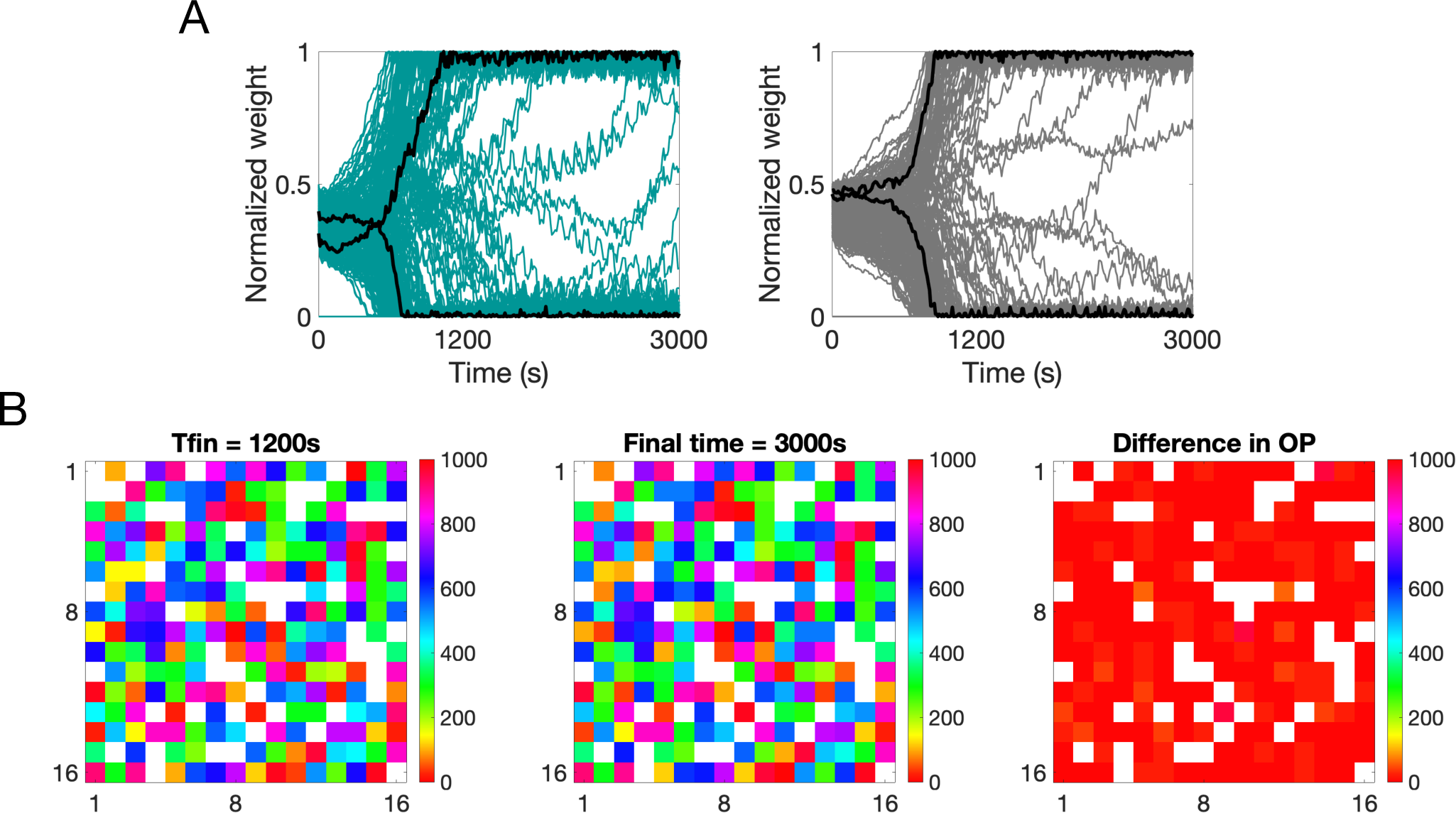

Supplement: S2 Fig — A: Plot of the change in feedforward weights to two sample cortical cells, one with GJs during the first phase of development (left, teal) and one without (right, gray), for 3000s of simulated time. The black curves highlight one weight that increased and one that decreased. Notice that by 1200s, we see a clear split of the weights and this cell has developed an OP. B: The resulting OP map for a simulation run for 1200s (left) and 3000s (middle) together with the difference in the OP between the two (right). The white squares indicate inhibitory neurons, which are not selective. The mostly-red plot shows that the OP for each neuron does not change much with longer simulation time. (TIF) [file pcbi.1007915.s002.tif]

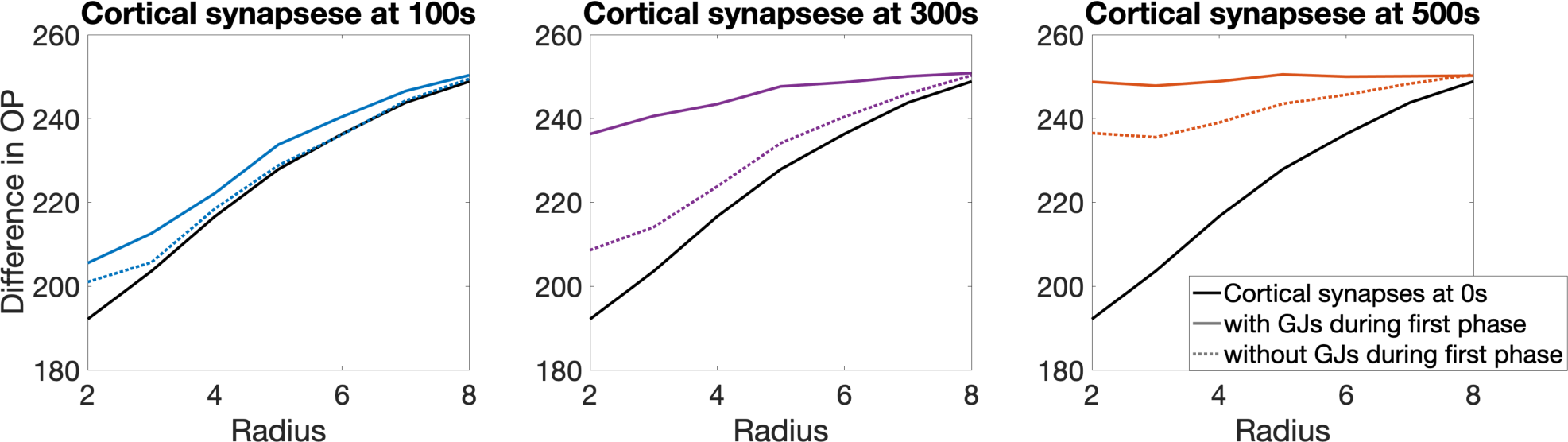

Supplement: S3 Fig — The panels show different start times of the recurrent cortical synapses increasing from left to right. The solid colored lines in each panel indicate those networks that contain GJ coupling during the first phase of development, while the dotted lines indicate those networks that do not. The black curve is for the case when cortical recurrent synapses and LGN feedforward synapses begin at the same time (independent of GJs). Notice that the measure is low (there is order in the OP map) for small radii, and increases with increasing radius, implying that cells share an OP at small distances, but not at large distances. Importantly, for the networks containing GJ coupling during the first phase of development, and for cases in which the feedforward synapses were allowed to learn for a sufficient amount of time while the GJs are present (300s and 500s), there seems to be little order for any value of the radius, see blue and orange solid lines in the first and third panels. For the same amount of feedforward learning, there is significantly more order in networks that did not contain GJ coupling, see dotted curves in all panels. (TIF) [file pcbi.1007915.s003.tif]
